# Arthroscopic Osteochondral Fragment Fixation of the Medial Femoral Condyle Using Knotless Suture Anchors

**DOI:** 10.1002/atn2.70106

**Published:** 2026-06-10

**Authors:** Molly N. Jones, Jeffton Pierre, Patrick Ryan, Clayton W. Nuelle, Steven F. DeFroda

**Affiliations:** ^1^ Univ. Missouri Dept. of Orthopaedic Surgery Missouri Orthopaedic Institute Columbia Missouri U.S.A.

## Abstract

Fixation of unstable osteochondral joint fragments is vital to long‐term joint function, but there are various repair techniques in the literature. This technical note describes a suture bridge technique performed arthroscopically that reduces the potential for future complications post operatively while stabilizing an osteochondral fracture fragment of the medial femoral condyle. The utilization of commonly available sutures and suture anchors to stabilize a well‐debrided osteochondral fracture fragment preserves native cartilage while restoring the articular surface.

## Graphical abstract

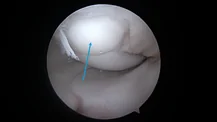

**VIDEO 1:** The accompanying video demonstrates a technique for the arthroscopic fixation of an osteochondral fracture fragment in the medial femoral condyle of the left knee using a suture bridge method. A 14‐year‐old female experienced left medial knee pain and dysfunction despite conservative management over approximately 3 years. Preoperative magnetic resonance imaging of her knee revealed an osteochondral lesion on the left medial femoral condyle with fluid signal underneath the fragment as well as cartilage heterogeneity and a fissure, findings consistent with an unstable fragment. Given her failure to improve with conservative treatment and the imaging confirmation of lesion instability, surgical fixation was deemed necessary to stabilize the fragment, relieve symptoms, and preserve the joint surface. Using a standard 2‐portal technique with a 30° scope, diagnostic arthroscopy was performed to begin the procedure. An unstable osteochondral fragment of the medial femoral condyle measuring 1.2 × 1.2 cm with surrounding frayed articular cartilage is then discovered. The frayed area and the base are debrided using a curette and shaver, along with an arthroscopic elevator to elevate the flap. The flap is microdrilled with a 1.25 mm K‐wire, and the wire is used to hold the fragment in a reduced position as needed. A 2.5 mm PushLock anchor is placed at the medial most apex of the defect and double loaded with suture. Both strands of suture are passed to a laterally based anchor within the notch. A row of more superficial sutures is retrieved crisscrossed over the prior ones and passed to a more inferior medial double row 2.5 mm PushLock anchor. Remaining loose cartilage is debrided and removed with a small full radius resector held at 90°. The intercondylar notch is microfractured, which aids in healing. Video content can be viewed at https://doi.org/10.1002/atn2.70106.

Acute osteochondral fractures (OCFs) of the knee, often involving the femoral condyles or patella, typically occur in adolescents after trauma or patellar dislocation.^1^ These injuries can be challenging to distinguish from osteochondritis dissecans (OCD) lesions, which classically affect the lateral aspect of the medial femoral condyle in youth.^1^ Regardless of etiology, an unstable osteochondral fragment or loose body in the joint is an indication for surgical intervention.^2^ Stable OCD lesions with intact cartilage are often managed nonoperatively in skeletally immature patients, but unstable, displaced, or persistently symptomatic lesions require surgical fixation to facilitate healing.^3^ Early identification of an osteochondral fragment is therefore critical, as its presence strongly warrants operative management to restore the articular surface and prevent long‐term joint damage.^2^

For larger osteochondral injuries, stable internal fixation is preferred over fragment excision, as it promotes fragment healing, maintains osteochondral viability, and helps prevent further chondral damage.^2^ Numerous fixation techniques have been described, including standard or headless compression screws, bioabsorbable screws and pins, meniscal darts, and suture‐through‐bone tunnel constructs.^1^ Metallic screw fixation provides strong compression and stability across the fracture site but carries risks of fragment fragmentation, intra‐articular hardware prominence, and often necessitates a second procedure for hardware removal.^2^ Bioabsorbable implants avoid retained metal hardware, yet their use has been associated with foreign‐body synovitis and delayed inflammatory reactions in the joint.^2^ Pure suture fixation techniques eliminate bulky implants, but they do not achieve the same compression or stability as rigid fixation, which can necessitate prolonged protected weight‐bearing and limited early motion postoperatively.^2^ These limitations have prompted development of alternative methods that provide secure fixation while minimizing hardware in the joint.

Knotless all‐suture anchor fixation has emerged as a technique to achieve stable osteochondral fragment fixation with minimal implant burden.^2^ This method can be performed entirely arthroscopically, avoiding an open arthrotomy and preserving the native osteochondral fragment. The small, flexible anchors require only minimal disruption of the underlying subchondral bone and are relatively sparing to the articular cartilage.^2^ By placing anchors directly beneath the fragment, the surgeon can compress the fragment onto its bed for anatomic reduction, and capturing the fragment with suture tension enhances its stability while reducing the risk of further fragmentation.^2^ Furthermore, the knotless anchor construct is low‐profile: it leaves no suture knots or prominent hardware within the joint that might cause irritation or require later removal.^2^ This technique thus provides secure internal fixation without any retained implants, facilitating early postoperative range of motion and obviating the need for secondary hardware‐removal surgery.^2^

## SURGICAL TECHNIQUE

Our surgical technique is shown in Video 1.

### Patient Preparation and Positioning

Arthroscopy is performed under general anesthesia. A pneumatic tourniquet is placed loosely on a well‐padded thigh. The patient is placed in a supine position with a lateral post and is then prepped and draped in a standard sterile fashion. Proper timeouts are utilized to verify the patient and the operative site.

### Diagnostic Arthroscopy

**FIGURE 1:**
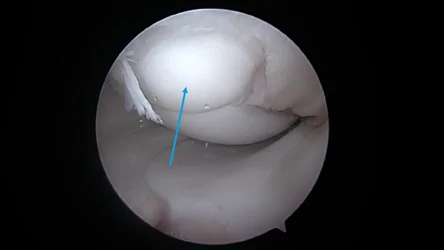
A 14‐year‐old female patient that failed conservative treatment underwent arthroscopic fixation of an osteochondral fracture (OCF) fragment of the left medial femoral condyle (MFC) with knotless suture anchors. This picture demonstrates the identified lesion of the left MFC viewing from the anterolateral portal with a 30° scope (Arthrex, Naples, FL). The lesion is identified with the blue arrow in the image.

The operative leg is exsanguinated to 250 mmHg. A 2‐portal technique is used, beginning with an anterolateral portal created for arthroscope placement and inflow and outflow. An anteromedial portal is then created via inside out technique and used primarily for instrumentation. A standard diagnostic arthroscopy is then performed using a 30° scope (Arthrex, Naples, FL), with the only abnormal finding in this case being an unstable osteochondral fragment of the medial femoral condyle measuring 1.2 × 1.2 cm. The lesion is surrounded with frayed loose articular cartilage (Figure 1).

### Arthroscopic Repair

**FIGURE 2:**
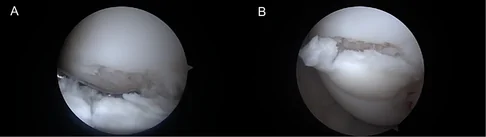
A 14‐year‐old female patient underwent arthroscopic fixation of an OCF fragment of the left MFC with knotless suture anchors. In this image, the 30° arthroscope (Arthrex, Naples, FL) is placed in the anterolateral portal and a probe is placed in the anteromedial portal. The lesion has been debrided of any loose fragments with a shaver, creating a healthy bed for the cartilage fragment. A shows the base of the lesion while retracting the fragment with the probe, while B shows the lesion now cleaned of any loose fragments.

**FIGURE 3:**
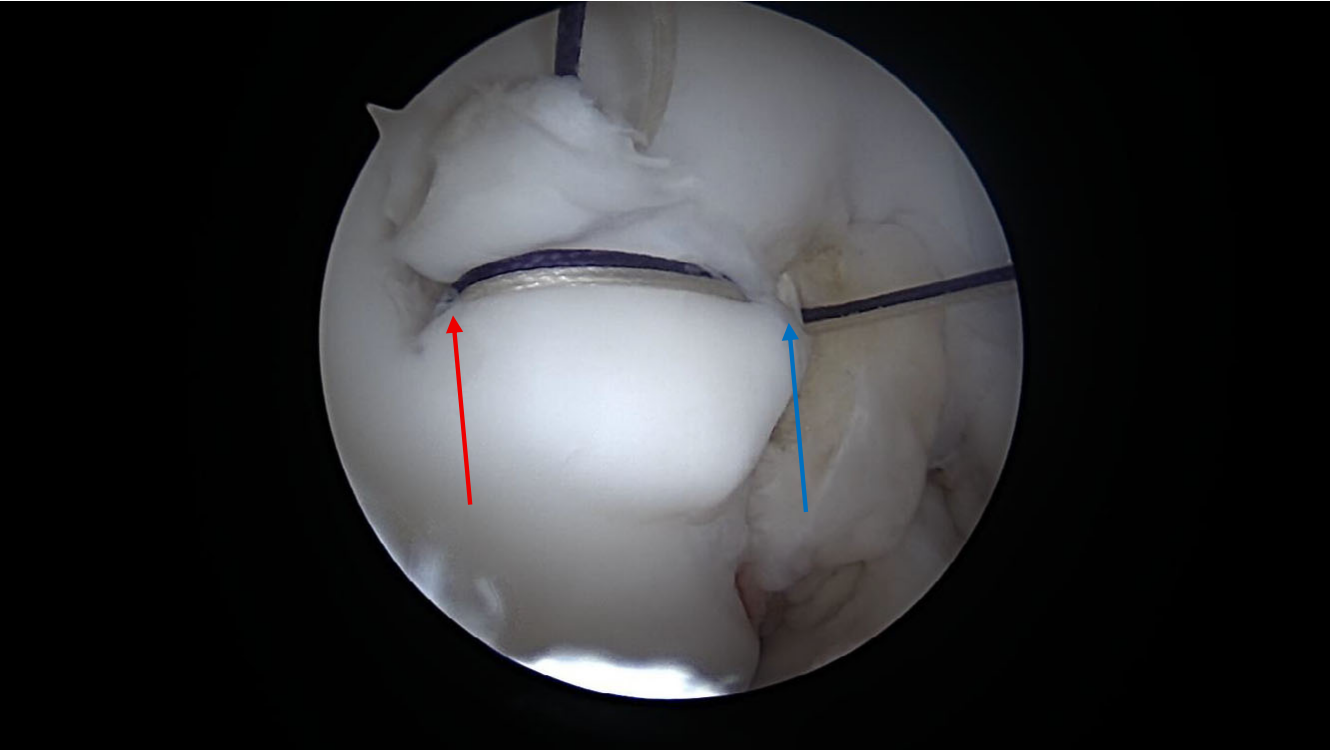
A 14‐year‐old female patient underwent arthroscopic fixation of an OCF fragment of the left MFC with knotless suture anchors. The placement of the first row of sutures is shown in this image via a 30° arthroscope (Arthrex, Naples, FL) in the anterolateral portal. The red arrow demonstrates a PushLock anchor (Arthrex, Naples, FL), initially placed in the medial aspect of the reduced fragment. This is double loaded resulting in total 4 suture strands. The first 2 strands are then retrieved and dunked into a second PushLock anchor (Arthrex, Naples, FL), identified by the blue arrow, in the superolateral aspect of the fragment, providing excellent initial compression over the fragment into its anatomic bed.

**FIGURE 4:**
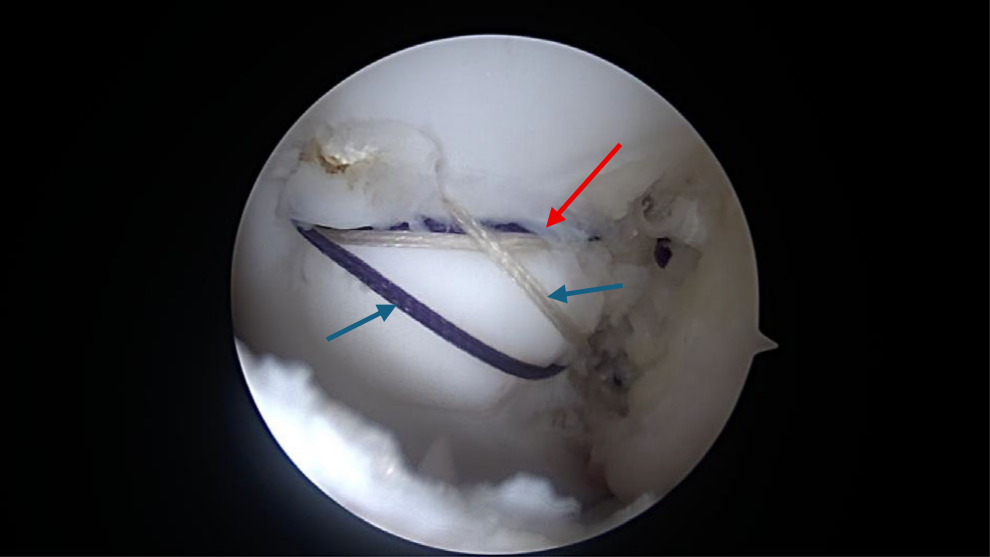
A 14‐year‐old female patient underwent arthroscopic fixation of an OCF fragment of the left MFC with knotless suture anchors. This image is the view from the anteromedial portal via a 30° scope (Arthrex, Naples, FL) demonstrating the final construct. Following the anterolateral PushLock fixation, the second pair of sutures from the medial based anchor are dunked into a third PushLock (Arthrex, Naples, FL) anchor placed in the posterolateral aspect of the fragment, identified by the blue arrows. The red arrow demonstrates the first suture pair placed into the anterolateral anchor.

Any fibrocartilage is then debrided with a shaver. The flap is now elevated with an arthroscopic elevator (Arthrex, Naples, FL), and the base of the lesion is debrided with a curette (Arthrex, Naples, FL) and a shaver (Arthrex, Naples, FL) (Figure 2). Bleeding bone is created to help promote healing. The base of the fracture fragment is micro drilled with a 1.25‐mm K‐wire, and then the wire is used to hold the fracture reduction as needed. A 2.5‐mm PushLock anchor (Arthrex, Naples, FL) is then placed at the medial most apex of the defect. This is double loaded with 2 sutures. The 2 strands of suture are then retrieved and passed to a laterally based anchor within the notch (Figure 3). The second set of sutures are retrieved crisscrossed over the prior ones and seated into a more inferior medial double row 2.5‐mm PushLock anchor within the notch (Figure 4). Any unstable cartilage is removed and debrided from the defect. Remaining loose articular cartilage can now be gently removed with a small full radius resector (Arthrex, Naples, FL) held at 90° to prevent over‐debridement and to achieve a smooth articular surface upon completion of the chondroplasty. The intercondylar notch is microfractured to promote healing. Pearls and pitfalls of this technique are listed in Table 1. The knee is irrigated with several liters of Ringers solution. The portals are closed with 3‐0 prolene.

**TABLE 1:** Pearls and Pitfalls of the Technique

**Pearls**	**Pitfalls**
• Restores articular surface of the weight‐bearing medial femoral condyle which may help prevent subsequent degenerative changes in the medial compartment • Allows for excellent compression of the osteochondral fragment • Preserves substantially more native cartilage within the fragment than techniques that involve screw fixation • Restoration of the medial femoral condyle congruity may help to decrease graft overload and subsequent failure • Procedure can be performed using common, likely readily available implants	• Improper anchor placement can compromise graft stability • Prominence of suture may result in shearing force on the articulating tibial cartilage • Compression across the osteochondral fragment may not be uniform and may be stronger directly deep to the crossing suture • Subchondral bone may largely be involved in the fragment which may compromise anchor fixation • Use caution when performing microfracture so as not to risk adequate fixation of the suture anchor

### Postsurgical Care

Standard sterile dressings and an Ace wrap for compression are applied. Postoperative instructions allow for the patient to be touch weight‐bearing in extension, and the patient is subsequently placed under OCD rehabilitation protocol.

## DISCUSSION

Published techniques highlighting the fixation of OCF fragments using minimally invasive methods are limited. Fixation techniques utilizing nonabsorbable screws preserve native cartilage, though iatrogenic cartilage damage from the screws is a risk.^4^ Additionally, 2 operative stages are required with the use of nonabsorbable screws, as screw removal in a second, later stage is necessary.^4^ Generally, hardware can also cause complications such as inflammation and foreign body reactions.^1^ Open reduction and internal fixation have been shown in the medial femoral condyle, although this is invasive and may be better suited for larger fragments.^5^

In order to minimize notable side effects and complication profiles associated with hardware fixation, techniques involving suture anchors have been of increasing interest. Such techniques aim to eliminate staged procedures and minimize the disruption of native cartilage without compromising fixation. There is evidence that the suture bridge technique for the fixation of OCD, OCFs, and chondral fragments is successful, with full union of up to 82.5% of lesions.^6^

Samitier et al. describe a similar one‐step technique using knotless anchors and interconnected suture sliding loops that shows promise for various anatomical locations.^1^ This technique does involve reshaping the fragment after removing it from the knee, which could lead to malposition on replacement.^1^ Vogel et al. describe a similar method using sutures in a bridge‐like fashion, although this procedure was not done completely arthroscopically.^7^ These techniques have the potential to treat OCF fragments in a way that restores function while reducing long‐term consequences such as chondral damage from hardware.^1^, ^7^

Our technique provides stability and creates an environment that promotes healing, while being done completely arthroscopically. This allows for a minimally invasive approach. Furthermore, this technique does not require completely removing the fracture fragment from the knee for debridement and cleaning. This may lead to less exposure that could potentially increase risk of infection. The major limitation of this technique is that it is likely highly dependent on the shape and severity of the fracture and therefore may not be applicable in all cases. Advantages and disadvantages of this technique are listed in Table 2.

**TABLE 2:** Advantages and Disadvantages of the Technique

** Advantages**	**Disadvantages**
• Only a 1‐step surgery • No metallic hardware, therefore, a lesser risk of further chondral damage or reactions • Can be done arthroscopically • Is likely applicable to multiple anatomical locations • Debridement and creation of bleeding bone allow for an environment supportive of healing • Multiple rows of suture promote fragment stability	• May be less applicable for large fragments • Potential for complications related to irritation from sutures

Further studies comparing outcomes for various methods of OCF fragments long‐term are necessary to determine the clinical utility of this method. Investigating time from injury to repair for techniques such as this could be of importance and may help define the time window in which success and healing is most likely.

## DISCLOSURES

The authors (P.R., C.W.N., S.F.D.) declare the following financial interests/personal relationships which may be considered as potential competing interests: P.R. reports a relationship with *Arthroscopy* that includes: board membership. C.W.N. reports a relationship with Arthroscopy Association of North America that includes: employment; Burkhart Research Institute for Orthopaedics that includes: board membership; *Arthroscopy* Journal that includes: board membership; other professional activities include: IP Royalties AO Foundation, Fiduciary officer at AO North America, American Academy of Orthopaedic Surgeons, American Orthopaedic Society for Sports Medicine; Other professional activities at Arthrex, Vericel, Guidepoint Global. S.F.D. reports a relationship with AO North America that includes: speaking and lecture fees; Stryker that includes: consulting or advisory; and *Arthroscopy* that includes: board membership; reports research support received for OREF, AANA, Arthrex, and Stryker; and is a committee member for AANA and AOSSM, unrelated to this article. The other authors (M.N.J., J.P.) declare that they have no known competing financial interests or personal relationships that could have appeared to influence the work reported in this paper.
